# International web survey shows high prevalence of symptomatic testosterone deficiency in men

**DOI:** 10.3109/13685538.2010.511325

**Published:** 2010-08-27

**Authors:** TOM R TRINICK, MARK R FENELEY, HUGH WELFORD, MALCOLM CARRUTHERS

**Affiliations:** 1 Department of Chemical Pathology, The Ulster Hospital, Belfast, United Kingdom; 2 Institute of Urology and Nephrology, University College Hospital, London, United Kingdom; 3 Centre for Men’s Health, London, United Kingdom

**Keywords:** AMS questionnaire, web survey, testosterone deficiency, diagnosis, treatment, diabetes, alcohol, vasectomy

## Abstract

**Introduction:**

Though the clinical significance of testosterone deficiency is becoming increasingly apparent, its prevalence in the general population remains unrecognised. A large web-based survey was undertaken over 3 years to study the scale of this missed diagnosis.

**Methods:**

An online questionnaire giving the symptoms characterising testosterone deficiency syndrome (Aging Male Symptoms – AMS – scale) was set up on three web sites, together with questions about possible contributory factors. Results. Of over 10,000 men, mainly from the UK and USA, who responded, 80% had moderate or severe scores likely to benefit from testosterone replacement therapy (TRT). The average age was 52, but with many in their 40s when the diagnosis of ‘late onset hypogonadism’ is not generally considered. Other possible contributory factors to the high testosterone deficiency scores reported were obesity (29%), alcohol (17.3%), testicular problems such as mumps orchitis (11.4%), prostate problems (5.6%), urinary infection (5.2%) and diabetes 5.7%.

**Conclusions:**

In this self-selected large international sample of men, there was a very high prevalence of scores which if clinically relevant would warrant a therapeutic trial of testosterone treatment. This study suggests that there are large numbers of men in the community whose testosterone deficiency is neither being diagnosed nor treated.

## Introduction

There is increasing recognition among doctors that testosterone deficiency is a common and important condition. As well as the key symptoms of loss of energy, drive, libido and impaired erectile function, there is often depression, memory loss and increased irritability which can impair work, home and social life.

This has also been causally linked to a wide range of medical conditions [1] including metabolic syndrome [2], diabetes [3], cardiovascular disease [4], osteoporosis [5], Alzheimer’s disease [6], depression [7], frailty [8] and even premature death [9].

An even more important point is that not only are there strong theoretical and epidemiological links to these disorders, but testosterone treatment has also been shown to be effective in the prevention and treatment of many of them, especially diseases linked with the recent epidemic of obesity such as metabolic syndrome, Type 2 diabetes [10] and coronary heart disease [11]. Also many of the conditions seen in aging populations world-wide which lead to frailty and disability can be prevented bu such treatment, making it an effective form of preventive medicine. In view of these benefits, it was decided to undertake a web-based survey of the prevalence of symptomatic testosterone deficiency.

## Methods

The standard English version of the 17-item Aging Male Symptoms (AMS) scale [12], which is well validated and widely used in research studies both to detect symptoms which might suggest testosterone deficiency syndrome (TDS) and monitor its treatment [13,14], was made available on the web-site of The Society for the Study of Androgen Deficiency (SSAD – Andropause Society) and two other Centre for Men’s Health web sites.

Additional questions were asked which were thought would give information about factors which might be related to androgen deficiency. As well as age, these included questions about whether the respondent had adult mumps, orchitis or other testicular problems, prostate operation or inflammation, persistent urinary infection, vasectomy, recent weight gain, diabetes or a high alcohol intake. For a representative sub-sample, demographic data on city and country of domicile and occupation were available.

On completion of the full questionnaire, the subject could immediately learn his total score and rating according to the standards for this scale: 17–26 none, 27–36 little, 37–49 moderate, over 50 severe. Given the high response rates to testosterone treatment of scores at these levels, moderate and severe scores were considered to represent testosterone deficiency, and the advice given to consult a physician.

Of the 13,861 respondents taking the online AMS questionnaire between August 2007 and January 2010 (28 months), 10,896 gave permission for their data to be analysed, and this was used in the study. The information from the full questionnaire from all three web-sites was down-loaded to an Excel spreadsheet for analysis, using PASW 18 statistics programme.

## Results

With the exception of the respondents to the SSAD site who gave a higher ‘Permission to use for analysis’ rate than either of the two commercial sites, i.e. was more trusted, the data were very similar, from all three web-sites, as shown by the mean age and total AMS scores, as well as in the more detailed analyses, two-thirds coming from the SSAD site (Table I). The combined data from the three sites are therefore used in this analysis.

**Table I:** Analysis of data from three web-sites.

	All data	SSAD	CMH	MHC
Number with permission	108,96	6885	1001	3010
Source of data	100%	63%	9%	28%
% Refusing permission	22%	17%	32%	26%
Mean age	52.0	51.7	51.8	52.5
Total AMS score	48.6	48.9	50.3	52.4

### Age and location of respondents

**Figure 1:**
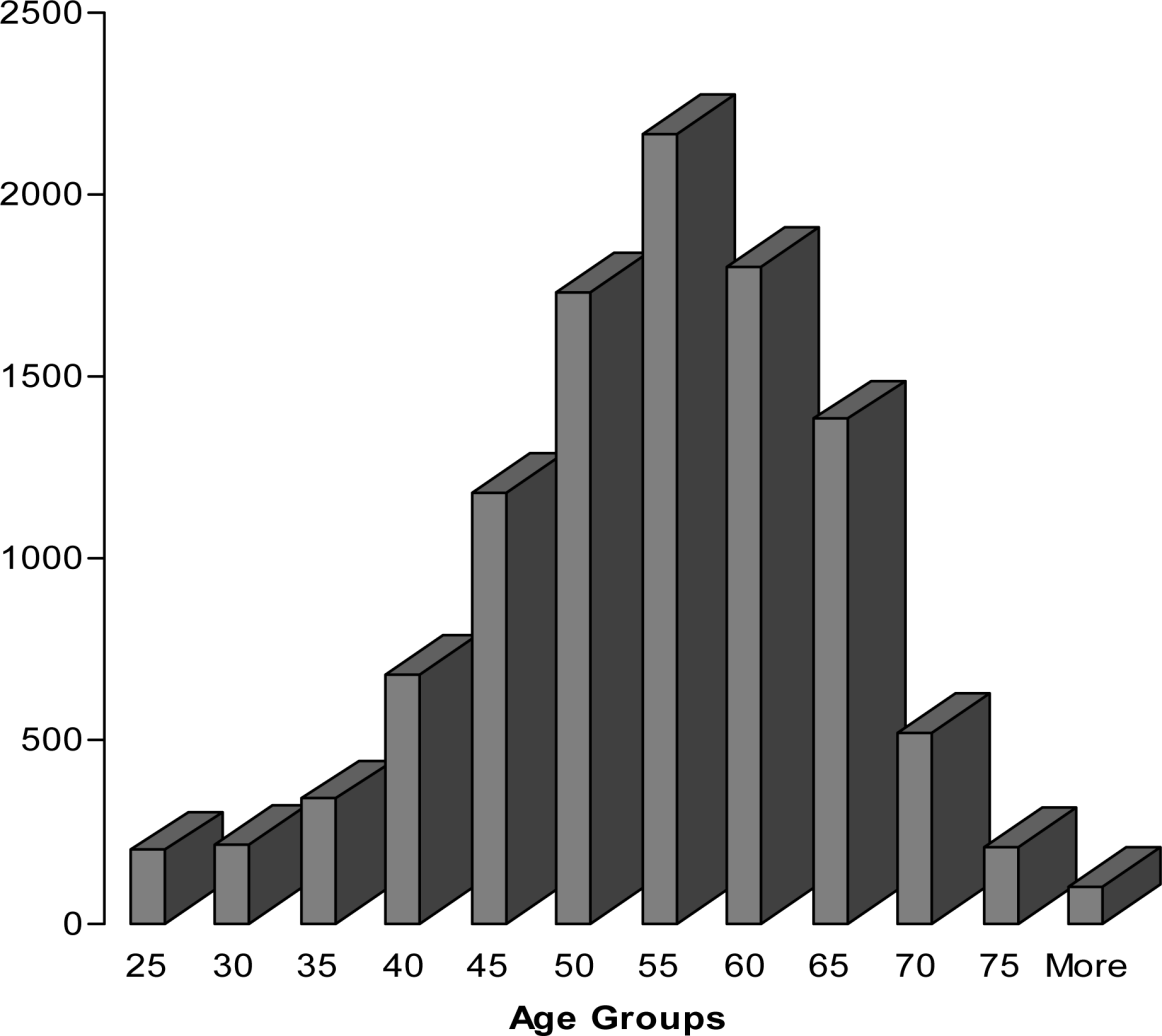
Age distribution of web AMS respondents.

The age range of the respondents was 16–89 (mean 52 years), which is close to the mean age of patients attending the Centre for Men’s health prior to treatment, and recognised as the time when these symptoms most commonly present as the ‘Andro-pause’ or ‘Male Menopause’ (Figure 1). Though symptoms of testosterone deficiency rarely present below the age of 30, and can be recognised even in the very elderly, the AMS scale, despite its name is not significantly age-related, unlike other commonly used scales [15].

While there may have been a few respondents in their 20s or earlier who were just experimenting with the AMS questionnaire, it is remarkable that because of spread of the normal distribution curve, 13% were below the age of 40, and another 27% in their 40s, i.e. 40% at ages when ‘Late Onset Hypogonadism’ is generally not recognised as occurring. However it has been found that the AMS scale can be used to measure and compare the health-related quality of life in those of less than 40 years of age on the basis of similar European normative values [16]. The surprisingly high prevalence of raised scores in the younger age groups may be due to the increasing impact of work stress in these age groups, which has been shown to decrease testosterone synthesis [17,18] and increase resistance to its action [19].

Seventy percent of respondents were from the UK, 10% from America, and the remainder from mainly English speaking countries all over the World. There was a wide scatter of occupations, and about 25% were retired as would be expected from the age distribution.

### Severity of symptoms

**Figure 2:**
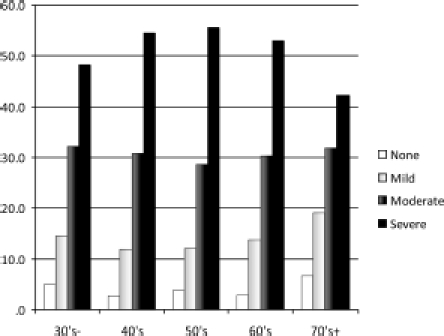
Severity of AMS symptoms in different age groups.

As rated by their total AMS scores overall 30.3% had ‘moderate’ and another 49.7% ‘severe’ symptoms, i.e. 80% having symptoms which gave them a high probability of having TDS and which would warrant a therapeutic trial (Figure 2). The proportion of ‘severe’ symptoms was slightly lower in the 30s and less and 70s and over age groups, peaking in the 50s, but this was balanced by raised ‘moderate’ scores in the former groups.

Of the individual questions, as might be expected, the three most highly rated questions were in the sexual subscale of the AMS questionnaire, i.e. decreases in ability and frequency of sexual activity, morning erections and libido.

The prevalence of related factors is described in the discussion section.

## Discussion

This was not a random survey of the general population, but was biased towards those searching the web for an explanation of symptoms they thought might be related to testosterone deficiency. Also there could be over-representation of younger and more computer literate age groups.

However, of this self-selected group of men who answered an online version of the widely studied and well validated AMS questionnaire, the results clearly show that a very large number of men identify with these classic symptoms of TDS. If they had been seen in a clinic setting where other causes of these symptoms could be excluded, as many as 80% might warrant a therapeutic trial of testosterone treatment, with a high probability of a positive response [20].

### Related factors

**Figure 3:**
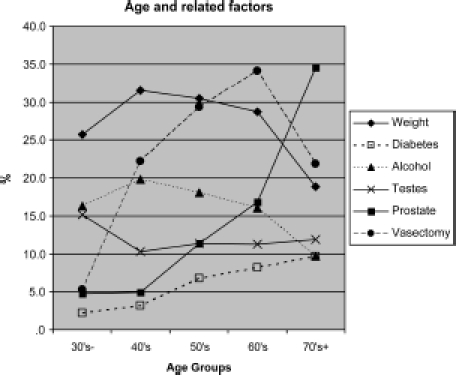
Additional factors reported in the different age groups.

In order of frequency, positive responses to the additional questions following those of the AMS scale (Figure 3) were as follows:

#### Recent weight gain (29% overall)

This was noted most frequently in the 40-and 50-years-old age groups. Whether this could be considered as a cause or a symptom is unclear, but this common complaint of patients presenting for testosterone treatment can be explained by the effect of testosterone deficiency on the multipotent stem cell [21]. This promotes differentiation from progenitor cells for muscle, bone and epithelium, towards the preadipocyte and adipocyte lineage. The resulting increase in adipocytokines contributes to the greater prevalence of metabolic syndrome, Type 2 diabetes, hypertension and circulatory diseases seen in obese individuals both in population surveys and in medical clinics. This has caused the adipocyte to be termed ‘The axis of evil’ [21].

#### Vasectomy (25% overall)

The reported frequency of this operation rose rapidly from 5% in the 30s age group, to a peak of 34% in the 60s group, falling away to the over 70s age group when vasectomy was less common.

This is thought to be about twice the frequency of this operation in the general population, and is a significant finding of this study. It is closely similar to the 24% found in the UK Androgen Study (UKAS) [22]. This could be because vasectomy is a marker for the ‘High-T’ men who are more aware of symptoms when their testosterone drops later in life. Alternatively, vasectomy could cause long-term damage to the testosterone-producing capacity of the testes many years after the operation for reasons yet to be explained [22].

#### Alcohol (17.3% overall)

This response to the question about ‘High alcohol intake’ rose to a maximum of 20% in the 40s, and is likely to have been an underestimate because the population is modest about reporting what it considers a reasonable intake in relation to the proven reduction in testosterone caused by alcohol, particularly with binge drinking. Also, unlike the liver, the testis has limited powers of regeneration, and high alcohol intake, even many years previously, can cause lasting damage to both spermatogenesis and testosterone production.

#### ‘Testicular problems and orchitis’ (11.4% overall)

While mumps orchitis has been described as the classic example of an infection causing an endocrine disorder, other viral infections such as glandular fever can also cause testicular damage and orchitis, as can a variety of sexually transmitted diseases. The reported prevalence of these testis-related conditions was as might be expected highest in the youngest group, but remained relatively constant at about 12% at other ages.

#### ‘Prostate operations and infections’ (5.6% overall) and urinary infections (5.2% overall)

As well as being stressful events likely to interfere with sexual activity, and directly or indirectly suppress testosterone production, benign prostatic enlargement has been linked with higher oestrogen levels, which especially in the obese individual can reduce androgen production.

Prostate related problems and urinary infections combined rose with age as would be expected to 17% in the 60s and 35% in the over 70s.

#### Diabetes (5.7% overall)

This rose from 2% in the 1930s to 10% in the 1970s. Together with obesity, it is recognised as one of the major predisposing causes of androgen deficiency [23]. Figures available for Ireland show a population prevalence for all cases of diabetes in 2005 of 5.4% in Northern Ireland and 4.7% in the Republic of Ireland. Taking into account population change and the linear rise in obesity, these figures are projected to rise to 6.3% in Northern Ireland and 5.6% in the Republic of Ireland by 2015 [24]. Significantly, low testosterone levels have been found in up to 50% of diabetics, and more importantly, the complications of this condition have been reduced by testosterone treatment [25].

### Symptomatic assessment

**Figure 4:**
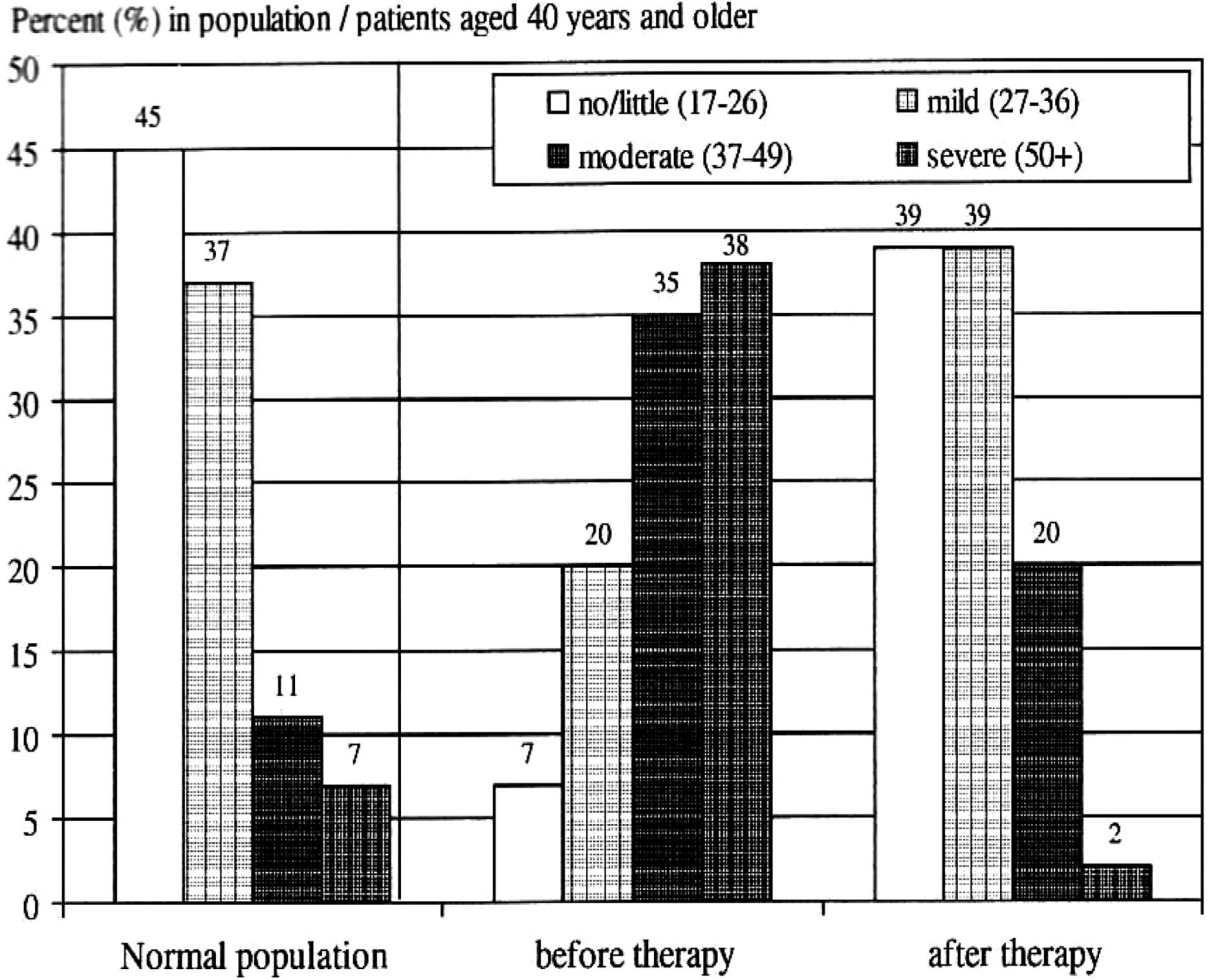
AMS Scores in normal population, and scores in patients over the age of 40 in patients in a urological clinic before and after 3 months testosterone treatment [20].

This level of symptoms at all ages is similar to that seen in two studies carried out in urological clinics in Germany where those men with moderate or severe AMS scores were treated with two different testosterone preparations, testosterone injections and gel, with equally good response rates [20,26] (Figure 4).

The uniform and sustained relief of these symptoms given by testosterone treatment has been confirmed by the experience over more than 15 years in 1700 men in the UKAS treated predominantly on the basis of their symptoms rather than androgen levels [19].

Though the AMS was not originally developed as a tool for diagnosing androgen deficiency, the majority of the questions in it overlap with the typical symptoms of TDS, which have been recognised and consistent since they were first described over 60 years ago by Dr. August Werner [27]. This questionnaire makes it possible to suspect TDS in the clinical setting where other causes of these symptoms can be excluded.

The paradox is that these characteristic symptoms of testosterone deficiency are very poorly correlated with total testosterone (TT) or other androgen levels in the blood. This can be explained by the concept of ‘androgen resistance’ [19]. As with insulin in maturity onset diabetes mellitus, there can be both insufficient production and variable degrees of resistance to the action of androgens operating at several levels in the body simultaneously, with these factors becoming progressively worse with aging, adverse lifestyle, other disease processes, and a wide range of medications. The mechanisms by which androgen deficiency acts can be considered at five different levels:

1. Impaired androgen synthesis or regulation.
2. Increased androgen binding.
3. Reduced tissue responsiveness.
4. Decreased androgen receptor activity.
5. Impaired transcription and translation.

This provides an explanation of why scores on a range of questionnaires listing symptoms of testosterone deficiency consistently fail to predict low androgen levels.

For example, a recent report on the AMS scale, states that ‘the total AMS score was not significantly associated with TT’ [28]. Similarly, using three questionnaires, including the AMS and Androgen Deficiency in the Adult Male – St Louis (ADAM) scales, no relationship was found between symptomatology and any of a battery of eight endocrine assays, including TT and free testosterone (FT), other than possibly age-related declines in dehydroe-piandrosterone (DHEA) and insulin-like growth factor 1 (IGF-1) [29]. Further, investigation of a group of 81 Belgian men aged 53–66 (mean 59) concluded ‘there was no correlation between AMS (total and subscales) and testosterone levels [30], while the same group in a study of 161 more elderly men aged 74–89 (mean 78) also showed no correlation between symptom scores and TT, FT, or bioavailable testosterone (BT)’ [31].

One of the studies most clearly highlighting the paradox dividing androgen deficiency symptom scales and laboratory measures is that of Miwa et al. in 2006, who found no correlation between the total and psychological, somatic or sexual domain scores of the AMS and serum levels of TT, FT, estradiol (E2), luteinizing hormone (LH), follicle stimulating hormone (FSH), dehydroepian-drosterone-sulphate (DHEAS), or growth hormone (GH) [32].

Because of this high sensitivity but low specificity of questionnaires to detect patients with low levels of androgens, the complexity of factors involved in androgen resistance, and the lack of validity of androgen assays [33], it seems logical to adopt the suggestion endorsed by Black et al. [34], which is where typical symptoms or conditions known to be related to androgen deficiency occur, that a 3-month therapeutic trial of testosterone treatment be given, providing it appears clinically justified.

This coincides with the emerging view that ‘An emphasis and reliance on serum T alone hinders the clinician’s ability to manage testosterone deficiency syndromes (TDS)’ [29]. Low total testosterone is just the tip of the iceberg of androgen deficiency, and is a poor marker of the underlying larger mass of symptoms and metabolic disturbances.

The high level of symptoms reported by these men in the web survey highlights the findings in a recently published study by one of the report’s authors ‘Time for International Action on Treating Testosterone Deficiency Symptoms’ [35]. This gives world-wide data showing that testosterone deficiency is the commonest hormonal disorder in men, but the least commonly treated. In the UK and all European countries, as well as Australia, Japan and Russia, of the 20% of men over the age of 50 who on a symptomatic basis can be rated as deficient in this hormone, less than 1% are being treated. The USA treats about 8%. This makes testosterone deficiency the most common endocrine disorder in men, and yet the least often diagnosed and treated.

Diagnosis and treatment of testosterone deficiency is economic and safe, and represents an important part of the preventive medicine of the future. This is not a condition which we can afford to leave untreated for either the health or welfare of an aging society.

In the light of the poor quality of life, energy, vitality and sexual function of the testosterone deficient man, as well as the associated conditions such as metabolic syndrome, diabetes, cardiovascular disease, and osteoporosis, how long can the medical community or health services afford to let men continue to go without this treatment?
